# X-ray phase-contrast microtomography of soft tissues using a compact laboratory system with two-directional sensitivity

**DOI:** 10.1364/OPTICA.487270

**Published:** 2023-06-30

**Authors:** Carlos Navarrete-León, Adam Doherty, Savvas Savvidis, Mattia F. M. Gerli, Giovanni Piredda, Alberto Astolfo, David Bate, Silvia Cipiccia, Charlotte K. Hagen, Alessandro Olivo, Marco Endrizzi

**Affiliations:** 1 Department of Medical Physics and Biomedical Engineering, University College London, Gower Street, London, WC1E 6BT, UK; 2 UCL Division of Surgery and Interventional Science, Royal Free Hospital, Rowland Hill Street, London, NW3 2PF, UK; 3 Stem Cell and Regenerative Medicine Section, Great Ormond Street Institute of Child Health, University College London, London, WC1N 1EH, UK; 4 Research Center for Microtechnology, Vorarlberg University of Applied Sciences, Hochschulstr. 1, 6850, Dornbirn, Austria; 5 Nikon X-Tek Systems Ltd, Tring, Herts, HP23 4JX, UK

## Abstract

X-ray microtomography is a nondestructive, three-dimensional inspection technique applied across a vast range of fields and disciplines, ranging from research to industrial, encompassing engineering, biology, and medical research. Phase-contrast imaging extends the domain of application of x-ray microtomography to classes of samples that exhibit weak attenuation, thus appearing with poor contrast in standard x-ray imaging. Notable examples are low-atomic-number materials, like carbon-fiber composites, soft matter, and biological soft tissues. We report on a compact and cost-effective system for x-ray phase-contrast microtomography. The system features high sensitivity to phase gradients and high resolution, requires a low-power sealed x-ray tube, a single optical element, and fits in a small footprint. It is compatible with standard x-ray detector technologies: in our experiments, we have observed that single-photon counting offered higher angular sensitivity, whereas flat panels provided a larger field of view. The system is benchmarked against known-material phantoms, and its potential for soft-tissue three-dimensional imaging is demonstrated on small-animal organs: a piglet esophagus and a rat heart. We believe that the simplicity of the setup we are proposing, combined with its robustness and sensitivity, will facilitate accessing quantitative x-ray phase-contrast microtomography as a research tool across disciplines, including tissue engineering, materials science, and nondestructive testing in general.

## 1. INTRODUCTION

X-ray microtomography has become an invaluable tool for the nondestructive volumetric imaging of samples for a wide variety of fields, ranging from medical sciences to metrology and manufacturing [1–3]. Despite continuous progress in x-ray microtomography technology, three-dimensional imaging of low-density samples, such as soft tissue specimens, remains challenging due to the weak x-ray attenuation contrast that is linked to low atomic number materials. X-ray phase-contrast microtomography (XPCµT) can overcome this limitation by exploiting also the phase shift experienced by the wave when traversing the object for generating image contrast [4], thus extending the applicability of microtomography to a broader range of samples and disciplines.

The technology for x-ray phase-contrast imaging is more demanding in comparison to what is needed for conventional x-ray imaging, specifically in terms of the required spatial and temporal coherence of the radiation [4]. While synchrotron radiation facilities can achieve high degrees of coherence, that is not often the case for laboratory-based systems, where the power density for x-ray generation is limited and a small footprint is required. In turn, this means that one has to choose between having a small or a powerful source, and working with monochromatic radiation often requires long exposure times. Many solutions have been identified and pursued over the past 25 years to overcome these limitations and facilitate the translation of x-ray phase-contrast imaging to a laboratory setting. Without compiling a complete history, here we summarize some of the milestones: microfocus source and crystal combination [5], free-space propagation with polychromatic radiation [6], Talbot–Lau interferometry [7–9], edge illumination [10,11], single-shot differential phase contrast and diffraction through spatial harmonic analysis [12], liquid metal jet sources [13], universal moiré effect [14], speckle-based imaging also in combination with liquid metal jet sources [15–18] and implicit tracking [19]; we refer to some excellent reviews on the topic for a more comprehensive list and discussion [20–26]. Notable laboratory-based XPCµT applications include imaging of soft-tissue specimens like lung [27,28], breast [29], heart [30], esophagus [31], brain [32], multimaterial phantoms [33–35], and for composites like carbon fiber-reinforced composite materials [36].

**Fig. 1.**
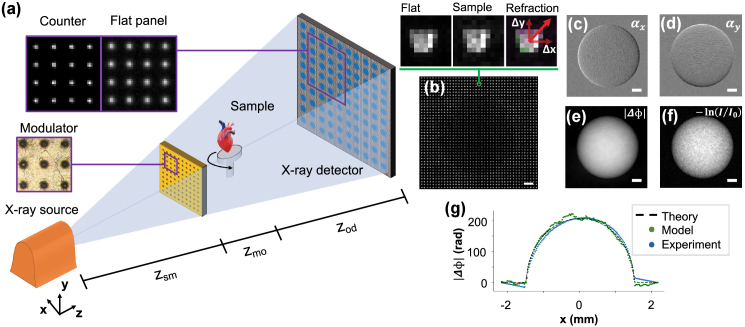
**System’s setup and working principle**. (a) Schematic of the laboratory set-up, including a sealed microfocus x-ray tube, the modulator, sample stage, and x-ray detector; (b) experimental image of a PMMA calibration sphere with an inset comparing beamlets without and with the sample in place; (c)–(d) effects of the presence of the sample are barely detectable by eye inspection; however, the dedicated data analysis algorithm reliably extracts refraction images. These are subsequently integrated leading to (e) phase images that offer superior signal-to-noise ratio to the (f) standard attenuation-contrast image. The refraction images are retrieved with a subpixel cross-correlation algorithm; we observe good agreement between the phase shift expected from theory, obtained through the numerical simulation, and from experimental data. All scale bars are 500 µm.

In this context, we have identified a solution for lab-XPCµT that has minimum requirements on the experimental setup. Our approach is based on a low-power, fixed target and sealed x-ray tube; it requires a single optical element (modulator), and it is compatible with flat-panel detectors as well as with single-photon counters. All of these components are readily available and require a minimal amount of maintenance. The modulator is fabricated through laser ablation of a tungsten foil and requires positioning accuracy of approximately 5–10 µm. The total length of the system is less than 1 m, making it one of the most compact for lab-XPCµT. Our approach shares similarities with a Shack–Hartmann wavefront sensor and in our work, it evolved from one-directional to two-directional sensitivity of beam tracking [37,38]. It can also be viewed as a highly parallelized version of a single-probe scanning system [39]. We call this approach two-directional beam tracking (2DBT). This solution offers a compact, robust, and cost-effective way of producing multicontrast three-dimensional images of the inner structure of samples, without compromising on angular sensitivity.

## 2. MATERIALS AND METHODS

### A. Model

The core element of the imaging system [Fig. 1(a)] is the structured illumination, obtained by spatially modulating the amplitude of the radiation field before it reaches the sample. Structuring the illumination in two dimensions enables tracking small changes in the position of each probe, i.e., measuring refraction along two orthogonal directions. It redefines sampling and spatial resolution of the imaging system. Sampling is no longer governed by the detector pixel pitch; it is instead defined by the distance between apertures in the modulator. The spatial resolution is redefined as being equal to or better than the width of the apertures in the modulator [40,41]. The main drawback of this approach is the sacrifice in flux available for imaging. We note, however, that this sacrifice does not affect dose efficiency because the radiation field is shaped before it reaches the sample. We also note that sampling is not necessarily limited by the pitch of the modulator: finer sampling can be achieved by recombining subsequent exposures, having moved either the modulator or the sample by a fraction of the pitch. We increase sampling by displacing the modulator by equally spaced subpitch increments. This requires a reference image for each modulator position; however, it preserves the conventional cone beam geometry and allows using standard three-dimensional reconstruction algorithms, even for the data sets acquired with higher sampling. Each probe is detected, and subsequently analyzed, independently. The x-ray intensity distribution of each probe is modeled through a series of convolutions,

(1) $$I_{0}(x,y)=(S*M*PSF)(x,y),$$

where ∗ denotes the two-dimensional convolution operator, S is the source intensity distribution, geometrically scaled by $-1+(z_{\text{sm}}+z_{\text{mo}}+z_{\text{od}})/z_{\text{sm}}$ , M is the transmission of the modulator (unity over a circle representing the aperture and zero elsewhere) geometrically scaled by $(z_{\text{sm}}+z_{\text{mo}}+z_{\text{od}})/z_{\text{sm}}$ , and PSF is the detector point spread function. Referring to Fig. 1(a), $z_{\text{sm}}$ is the distance between the source and the modulator, $z_{\text{mo}}$ is the distance between the modulator and the axis of rotation, and $z_{\text{od}}$ is the distance between the axis of rotation and the detector.

When a sample is in place, the intensity distribution of each probe is attenuated and shifted by the local properties of the sample,

(2) $$I(x,y)=tI_{0}(x-\Delta x,y-\Delta y),$$

where t is the transmission through the sample (at the position of the probe) and Δx and Δy are the probe displacements on the detector plane due to the refraction induced by the sample. These shifts are measured by comparing the probe intensity distributions with and without the object [Fig. 1(b)]. The transmission is calculated as the ratio of the total intensity in the probe,

(3) $$t=\frac{\sum I(x,y)}{\sum I_{0}(x,y)},$$

where sum operates over all the pixels illuminated by a single probe [Fig. 1(b), zoom-ins]. For the retrieval of the displacements Δx and Δy , we used a subpixel cross-correlation algorithm [42]. These displacements are related to the refraction angle and to the object-to-detector distance, $z_{od}$ , by

(4) $$\alpha_{x}(x,y)={\text{tan}}^{-1}\left(\frac{\Delta x}{z_{od}}\right),\;\alpha_{y}(x,y)={\text{tan}}^{-1}\left(\frac{\Delta y}{z_{od}}\right).$$

By linking the two refraction images to the orthogonal gradients of the phase shift (Fig. 1, panels c and d ) we obtain

(5) $$\alpha_{x}(x,y)=\frac{1}{k}\Delta \Phi_{x}(x,y),\;\alpha_{y}(x,y)=\frac{1}{k}\Delta \Phi_{y}(x,y),$$

where k is the wavenumber.

The phase shift of the wavefront ΔΦ(x,y) [Fig. 1(e)] can be obtained from the following expression:

(6) $$\Delta \Phi (x,y)=F^{-1}\left[\frac{F[\Delta \Phi_{x}+i\Delta \Phi_{y}](u,v)}{2\pi i(u+iv)}\right]\;(x,y),$$

where (u,v) represent the reciprocal space coordinates to (x,y) . This expression can be obtained using the Fourier derivative theorem as follows [43]:

(7) $$\begin{array}{ll} & F[\Delta \Phi_{x}+i\Delta \Phi_{y}](u,v)\\ & \;=\int \int_{-\infty }^{\infty }(\Delta \Phi_{x}+i\Delta \Phi_{y})\cdot e^{-2\pi i(ux+vy)}dxdy\\ & \;=2\pi i(u+iv)\cdot F[\Delta \Phi (x,y)]. \end{array}$$

A better signal-to-noise ratio can be seen by direct visual comparison with the attenuation-image contrast of the same phantom [Fig. 1(f)]. The retrieved quantities t and ΔΦ are line integrals along the photon path of two physical properties of the sample, the linear attenuation coefficient (μ) and the real part of the refractive index (δ) ,

(8) $$-\text{ln}\;t(x,y)=\int_{o}\mu (x^{\prime },y^{\prime },z^{\prime })dz,$$

(9) $$-\frac{\Delta \Phi (x,y)}{k}=\int_{o}\delta (x^{\prime },y^{\prime },z^{\prime })dz.$$

These relationships allow one to obtain volumetric reconstructions of μ and δ from projections taken at different viewing angles using the standard algorithms for tomography.

### B. Experiments

The x-ray system features a fixed W-target microfocus Hamamatsu x-ray source (L12161-07), which we run at 40 kVp and 10 W. The modulators were manufactured with a laser ablation procedure from readily available 100-µm-thick tungsten foils (Goodfellow). Two pitches, 50 and 100 µm, were tested. The apertures have a conical shape with diameters of ∼15µm and ∼30µm in the front and back apertures, respectively; the narrower aperture was facing the source.

Three detectors were tested: i) Pixirad-2/PIXIE-III photon counter with a 650 µm CdTe sensor (98% detection efficiency up to 50 keV), 62 µm pixel pitch and a $50\times 32\;{mm}^{2}$ active area; ii) MerlinX photon counter (Quantum Detectors) with a Medipix 3RX chip (256×256) , a 500 µm Si sensor and 55 µm pixel pitch; and iii) Hamamatsu flat panel (C9732DK-11) with directly deposited CsI scintillator, 50 µm pixel pitch and $12\times 12\;{cm}^{2}$ active area. Single-photon-counting detectors were chosen in view of achieving the highest angular sensitivity, whereas the flat panel was employed to allow for a larger field of view.

The geometry was defined as follows: $z_{\text{sm}}=(150,150,140)\;\;mm$ , $z_{\text{mo}}=(30,30,25)\;\;mm$ , and $z_{\text{od}}=(680,580,535)\;\;mm$ , for the Pixirad, MerlinX and Hamamatsu detectors, respectively.

To characterize the system and fine-tune the quantitative retrieval algorithm, a test phantom consisting of a poly(methyl methacrylate) (PMMA) calibration sphere (Goodfellow, ⊘=3.18±0.05mm ) was used. For the experimental assessment of spatial resolution, we used a phantom composed of soda-lime glass microspheres (Fischer Scientific, monodisperse, 50 µm diameter) deposited on a kapton substrate. The detector was the Pixirad with the 100 µm-pitch modulator and noise threshold set at 10 keV. A total of 16×16frames were acquired for both phantoms, with a sampling step in both x and y directions of 6.25 µm and 1 s exposure time per frame.

The angular sensitivity was quantified on planar images of a phantom composed of soda-lime glass microspheres (Fischer Scientific, monodisperse, 50 µm diameter) embedded in wax and polyethylene foam. Images were acquired at 4×4positions , with x and y steps of 12.5 µm. The detector was the MerlinX with the 50 µm-pitch modulator and noise threshold set at 5 keV. For each step, 256 frames of 0.25 s exposure time were acquired to study the angular sensitivity as a function of exposure time. The angular sensitivity was measured in an area without the sample by calculating the mean and standard error of the standard deviation of the measured refraction angles [44], nine different windows of 10×10pixels each were used. This approach quantifies the dispersion of noise in the refraction image, in turn indicating what is the minimum refraction angle that would be detectable by an imaging system.

The ability of the system to measure μ and δ in three dimensions was assessed with a multimaterial phantom composed of three spheres (Goodfellow) in a plastic straw. The materials were polypropylene (PP), polystyrene (PS) and PMMA with diameters: ${⊘}_{PP,PMMA}=3.18\pm 0.05\;mm$ , ${⊘}_{PS}=3.5\pm 0.1\;mm$ . The detector was the Pixirad with the 100 µm-pitch modulator and noise threshold set at 8 keV. We acquired 900 projections over $360^{\text{∘}}$ with 1 s exposure time per projection. A full rotation was acquired for each modulator position, giving a total of 8×8scans with sampling every 12.5 µm in x and y .

The performance of the system for soft-tissue imaging was demonstrated with two animal-derived small organs; a heart and an esophagus, extracted from a 300 g Sprague–Dawley rat and a 3 kg piglet, respectively. The heart sample was obtained from the UCL Biological Services Unit (BSU), while the esophagus from piglets was procured from JSR Genetics Ltd, a Home Office-approved supplier. Animals were euthanized via Schedule 1 methods, and sample sizing has been implemented following the NC3R principles. The organs were prepared for imaging through paraformaldehyde (PFA) fixation, followed by critical point drying (as described in previous work [31]). For the piglet esophagus, we acquired 900 equally spaced projections over $360^{\text{∘}}$ with 1 s exposure time using the Pixirad detector. The 100 µm-pitch modulator was stepped by 12.5 µm in x and y for a total of 8×8scans and an exposure time of 900×8×8×1s=16h . For the rat heart, we acquired 2000 equally spaced projections over $360^{\text{∘}}$ with 1.2 s exposure time using the Hamamatsu flat panel. The 50 µm-pitch modulator was stepped 4×4 times on a grid with 12.5 µm displacements in x and y . The total exposure time was 2000×4×4×1.2s=10.7h .

**Fig. 2.**
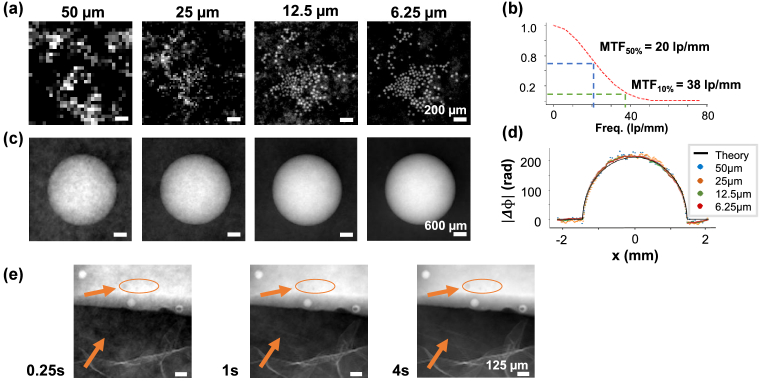
**System characterisation and benchmarking**. (a) Resolution and (c) accuracy of phase retrieval, as a function of sampling, in images of the monodisperse soda-lime glass microspheres on Kapton substrate and the PMMA calibration sphere, respectively; (a) the ability to visualize small details depends critically on a fine sampling and is mainly defined by the width aperture in the modulator. (b) The MTF matches well a real-space full width at half- maximum of 16 µm. (c) Phase is retrieved accurately in all cases, with higher noise linked to coarser sampling. This can also be seen in the (d) line profiles plot, where the phase shifts retrieved in the four experimental cases are compared to the theoretical phase shift. (e) Images of the angular sensitivity phantom showing microspheres and air bubbles embedded in wax in the upper half, and polyethylene foam in the lower half. Longer exposure times lead to better signal-to-noise ratios. For example, details in the foam and small void inclusions (indicated by arrows) become increasingly more visible. Exposures as short as 250 ms are sufficient for robust phase integration.

All reconstructions were performed using the Feldkamp–Davis–Kress algorithm implementation in CUDA of the Astra Toolbox [45]. The volume rendering presented in Fig. 5 were obtained using the Avizo software (Thermo Fischer Scientific).

## 3. RESULTS AND DISCUSSION

Figures 2(a) and 2(c) report the resolution phantom and the PMMA calibration sphere at different sampling steps, ranging from 50 to 6.25 µm. The positioning of the modulator has to be accurate and repeatable in comparison to these numbers, namely, approximately half of the aperture width, which is compatible with most micropositioning technologies. While the phase shift of a large object is correctly retrieved in all cases, the ability to represent finer details and distinguish between adjacent microspheres critically depends on the sampling step. The modulation transfer function (MTF) of the system at 6.25 µm sampling pitch is shown in Fig. 2(b), with 50% at 20 lp/mm and 10% at 38 lp/mm. The MTF was obtained as the Fourier transform of the PSF, which was experimentally evaluated against the images of a well-known object [46]. It matches well with the expectation from a real-space full width at half-maximum of 16 µm, comparable to the aperture size in the modulator. Figure 2(d) shows four line plots extracted from the phase images in panel 2(c) against the theoretically expected phase shift. There is good agreement between the phase shifts measured with different sampling steps and theory, with higher noise linked to lower sampling. The decrease in noise that is linked to progressively finer sampling is also visible by visual comparison of the four images in panel 2(c).

**Fig. 3.**
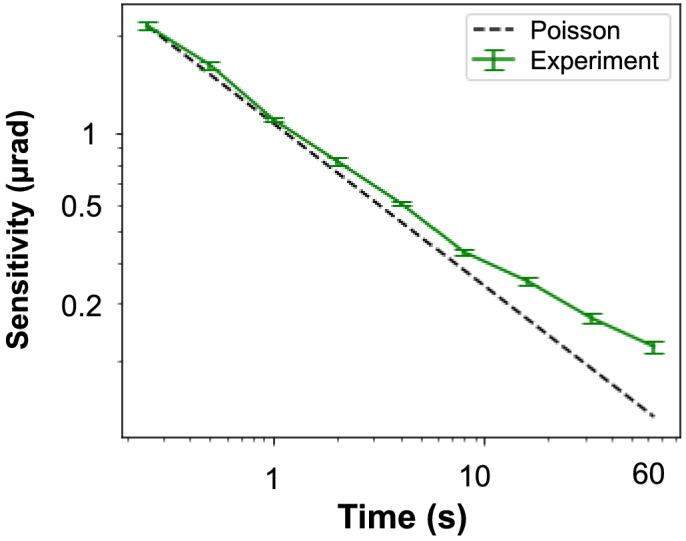
System angular sensitivity trend compared to quantum-limited ideal system, MerlinX detector, and 50 µm-pitch modulator.

The angular sensitivity depends on the exposure time and closely follows the trend expected from the Poisson statistics, proportional to the power of −1/2 (Table 1 and Fig. 3). This indicates that the main limitation in the phase retrieval process is photon statistics. The measured angular sensitivity starts departing from a perfect quantum-limited system for integration times approaching 10 s. This is due to environmental instabilities, including temperature drifts and vibrations, which become noticeable only with longer exposures. The integrated phase images of the sensitivity phantom are presented in Fig. 2(e). Smaller details emerge from the background noise as the exposure time is increased. We report submicroradian sensitivities starting at 2 s of exposure time and down to a minimum of 220±10nrad with 64 s per frame. These values are obtained with a single-exposure image, thus with a resolution limited by the modulator pitch at 50 µm. With the methods described earlier, the resolution can be increased up to approximately 16 µm while maintaining the same angular sensitivity and increasing the total exposure time. The angular sensitivity with the Hamamatsu flat panel was slightly worse, $(\sigma (\alpha_{x}),\sigma (\alpha_{y})=(1.96\;\pm \;0.05,\;2.10\;\pm \;0.05)\;\text{µ}rad$ and $(\sigma (\alpha_{x}),\sigma (\alpha_{y}))=(1.31\;\pm \;0.04,\;1.41\;\pm \;0.02)\;\text{µ}rad$ for 1 s and 2 s exposure times, respectively. However, the field of view was extended to 28mm×28mm from the 3.2mm×3.2mm achievable with the MerlinX. Many factors contribute to defining the angular sensitivity of a given system’s configuration, including detection efficiency, PSF, and stability during exposure. The development of a comprehensive model for angular sensitivity is underway and beyond the scope of this work. To position our system in the landscape of existing solutions for lab-based XPCµT, we report in Table 2 a summary of imaging system parameters from the literature, including source power, spatial resolution, system dimensions, and sensitivity. In comparison with EI and the first GBI systems, our approach achieves comparable angular sensitivity with a low-power source. The highest sensitivity was achieved with GBI [51] and the most powerful x-ray source, at the lowest spatial resolution. A more recent lab-based implementation of GBI [52] achieved high sensitivity at high resolution, and required fine-pitch gratings with a high aspect ratio. SBI [15] also reports high sensitivity and requires a large footprint and specialized source technology.

**Table 1.** System Angular Sensitivity for Different Exposure Times, MerlinX Detector, and 50 µm-pitch Modulator

Exp Time (s)	$\sigma (\alpha_{x})(\text{µ}rad)$	$\sigma (\alpha_{y})(\text{µ}rad)$
0.25	2.14±0.05	2.33±0.07
0.5	1.62±0.04	1.66±0.03
1	1.10±0.02	1.19±0.03
2	0.82±0.02	0.85±0.02
4	0.61±0.01	0.65±0.01
8	0.43±0.01	0.46±0.01
16	0.35±0.01	0.34±0.01
32	0.27±0.01	0.31±0.01
64	0.22±0.01	0.22±0.01

**Table 2.** Comparison of Laboratory-based XPCµT Systems in the Literature

Reference	Technique	Resolution	Sensitivity	Scan Time (s)	X-ray Spot Size	Source Power	Dimension
Diemoz *et al.* [47]	EI	12 or 66.8 µm	270 nrad	112 or 14 s	70 µm	875 W	200 cm
Havariyoun *et al.* [48]	EI	10 or 79 µm	230 nrad	384 or 24 s	70 µm	400 W	100 cm
Revol *et al.* [49]	GBI	104 µm	110 nrad	80.4 s	$1\times 1\;{mm}^{2}$	250–1000 W	140 cm
Thuring *et al.* [50]	GBI	4.1–7.1 µm	250–550 nrad	128 s	5–10 µm	4–12 W	20–33 cm
Birnbacher *et al.* [51]	GBI	170 µm	5 nrad	275 s	$132\times 226\;\text{µ}m^{2}$	2.8 kW	230 cm
Villa-Comamala *et al.* [52]	GBI	21.5 µm	45 nrad	250 s	10 µm	60 W	90.4 cm
Zanette *et al.* [15]	SBI	84 µm	240 nrad	300 s	$7.8\times 8.7\;\text{µ}m^{2}$	30 W	300 cm
Quénot *et al.* [19]	SBI	36 µm	5 µrad	600 s	4 µm	-	53 cm
Our setup with MerlinX	2DBT	15 or 50 µm	345 nrad	256 or 16 s	5–20 µm	10 W	70–86 cm

**Fig. 4.**
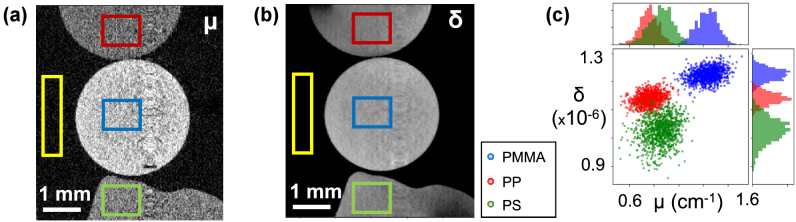
**Material identification**. Longitudinal slices of (a) μ and (b) δ for the multimaterial phantom; (c) the ability to separate the three materials is greatly improved by the simultaneous availability of the two contrast channels.

Tomographic reconstructions of μ and δ of the multimaterial phantom are presented in Figs. 4(a) and 4(b). The lower noise of phase tomography is apparent. This can be seen quantitatively in the histograms in panel 4(c), where the values extracted from the boxes highlighted in colors are compared. Phase contrast allows for a better separation of PP and PS with respect to attenuation contrast, and the simultaneous availability of both contrast channels improves material separation even further. Reconstruction artifacts, running vertically across both coronal planes in μ and δ images, are visible. They are linked to ring artifacts visible in the transverse plane slices, which were not correct for with dedicated filters. These artifacts were excluded from the regions of interest used for quantitative analysis. Measured values are compared against theoretically expected ones [53] in Table 3. Effective energies vary slightly for δ and μ as expected [54]; however, the range of values is very close to the mean energy of the x-ray spectrum (15.6 keV). The contrast-to-noise ratio (CNR) is calculated as

(10) $$CNR=\frac{\mu_{m}-\mu_{air}}{\sigma_{air}},$$

where $\mu_{m}$ is the linear attenuation coefficient of each material and $\sigma_{air}$ is the standard deviation of the background. An analogous formula is used for δ . CNRs were found to be substantially higher for phase tomography.

**Table 3.** Experimental and Theoretical μ and δ of the Phantom Materials, along with Effective Energies and CNRs Measured with Respect to the Background [yellow box in Figs. 4(a) and 4(b)]^*a*^

Material	$\mu_{exp}\;({cm}^{-1})$	$\mu_{theo}\;({cm}^{-1})$	$E_{\mu }\;(\text{keV})$	CNR of μ	$\delta_{exp}\;(\times 10^{-6})$	$\delta_{theo}\;(\times 10^{-6})$	$E_{\delta }\;(\text{keV})$	CNR of δ
PP	0.76±0.06	0.76	13	4.1	1.14±0.01	1.14	14	31.6
PMMA	1.23±0.06	1.23	15	7.1	1.22±0.01	1.22	15	34.0
PS	0.86±0.06	0.85	14	4.7	1.03±0.01	1.03	15	28.4

^*a*^ Good agreement is observed on both attenuation- and phase-contrast channels with expectations from theory. Phase tomography offers consistently higher CNRs.

**Fig. 5.**
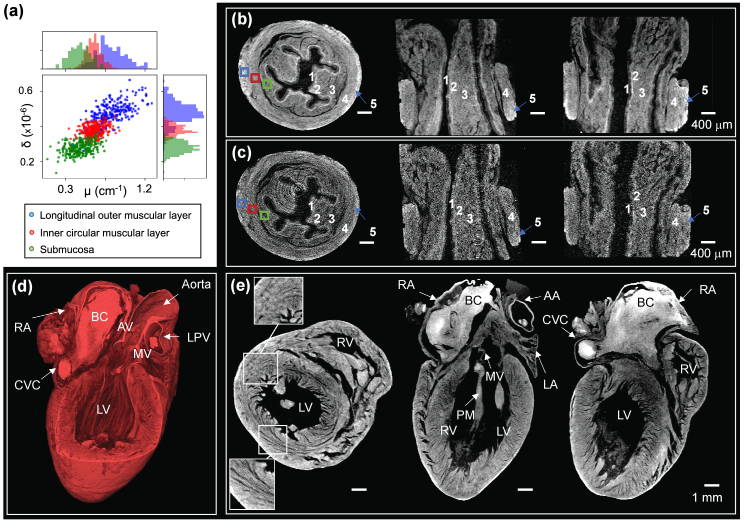
**XPCµT** **of (a)–(c) a piglet esophagus and a (d)–(e) rat heart;** Piglet esophagus: (a) μ and δ histograms extracted from (b) phase contrast and (c) conventional attenuation-contrast microtomography (axial, longitudinal, and coronal slices from left to right). The numerical labels correspond to five different tissue layers that were identified in the phase-contrast channel: (1) epithelium, (2) lamina propria, (3) submucosa, (4) inner circular muscular layer, and (5) longitudinal outer muscular layer. These layers are hardly if at all visible in the images of panel (c). For the rat heart: (d) volume rendering of the phase-contrast microtomography (e) and its axial, longitudinal, and coronal slices (from left to right). The following structures were identified: the LPV, the aorta, the AV, the AA, the MV, the LV, the RV, the PMs, the RA, and the CVC. We note the presence of a BC within the RA. The circumferentially oriented fibers in the mid-miocardium and the longitudinally oriented fibers closer to the outer epicardium wall can also be observed, as highlighted by the two zoom-in insets in panel (e).

Lab-XPCµT of the animal-derived small organs are presented in Fig. 5. The volume rendering and coronal, longitudinal, and axial slices of both samples are shown. Access to the full volume provides structural information of the specimens, which are represented with isotropic spatial resolution of approximately 16 µm.

For the piglet esophagus [Figs. 5(a)–5(c)], we identified five different tissue layers in the phase reconstructions as labeled in the images: (1) epithelium, (2) lamina propria, (3) submucosa, (4) inner circular muscular layer, and (5) longitudinal outer muscular layer. These tissue layers are poorly, if at all, visible in the attenuation-contrast reconstructions. Figure 5(a) reports the same principle previously shown for material identification in Fig. 4, now applied to three small volumes located in different tissue layers. Increased separation can be observed in phase contrast (δ) , and the simultaneous availability of both μ and δ representations vastly improves the ability to separate the layers. For the rat heart [Figs. 5(d) and 5(e)], lab-XPCµT allowed to visualize the orientation of microfibers in the muscle bundles and to identify the following structures: the left pulmonary vein (LPV), the aorta, the aortic valve (AV), the aortic arch (AA), the mitral valve (MV), the left ventricle (LV), the right ventricle (RV), the papillary muscles (PMs), the right atrium (RA), and the caudal vena cava (CVC). We note the presence of an iron-rich blood clot (BC) appearing brighter within the RA. The insets in the axial slice show the circumferentially oriented fibers in the mid-miocardium (line-like structures) and the longitudinally oriented fibers closer to the outer epicardium wall (point-like structures). The tomography data sets were acquired in a step-and-shoot fashion, which carried overheads. Without optimization, the total acquisition times were in the range of approximately 64 h. This mismatch is only due to a need for optimizing the sequences of positioning and detector exposure. Work is currently underway to implement fly-scans for bringing the data acquisition time much closer to the actual exposure times. This work focuses on XPCµT in a laboratory setting, and we reported only regarding the retrieval and reconstruction of two contrast channels, namely, attenuation and phase. We note, however, that the imaging system here proposed is compatible with x-ray dark-field imaging, and the approach presented here can be extended to diffuse dark-field imaging in two and three dimensions.

## 4. CONCLUSION

We presented a compact and cost-effective system for performing XPCµT in a laboratory setting. The system is based on a single modulator and a low-power sealed x-ray tube and is compatible with a range of readily available detectors. We report submicrometer angular sensitivity starting at 2 s of exposure time at 50 µm spatial resolution and 32 s at 16 µm spatial resolution, and a maximum angular sensitivity of 220 nrad in 64 s at 50 µm spatial resolution. These results are compatible with the state of the art and were achieved with a simple and compact setup. The proposed approach is quantitative, as validated through a phantom composed of known materials, and flexible in terms of spatial resolution and sampling. By introducing the amplitude modulator, sampling and resolution are driven by the structure of the illumination and can be tuned to the requirement of the sample by adapting the data acquisition strategy. The potential of the system for soft-tissue imaging was demonstrated on two biological specimens prepared without staining: five different tissue layers were identified in a piglet esophagus, and the orientation of microfibers within the myocardium of a rat heart was visualized. We believe that the concurrent simplicity, sensitivity, robustness, compactness, and efficiency of our approach will be instrumental in making XPCµT more accessible and available to a wider community.

## Data Availability

Data underlying the results presented in this paper are not publicly available at this time but may be obtained from the authors upon reasonable request.
